# Supplementary material for: A Collagen-Mimetic Organic-Inorganic Hydrogel for Cartilage Engineering
Source: Gels. 2021 Jun 15;7(2):73. doi: 10.3390/gels7020073 (PMC8293055; doi:10.3390/gels7020073)
Supplement: Supplementary file 1 [file gels-07-00073-s001.zip › gels-1248430-supplementary.pdf]

Supplementary materials

A Collagen-Mimetic Organic-Inorganic Hydrogel for Cartilage Engineering

Laurine Valot [1,2,†], Marie Maumus [3,4,†], Luc Brunel [1], Jean Martinez [1], Muriel Amblard [1], Danièle Noël [3,*], Ahmad Mehdi [2,*] and Gilles Subra [1,*]

1 IBMM, Univ Montpellier, CNRS, ENSCM, 34095 Montpellier, France; laurine-valot@laposte.net (L.V.); luc.brunel@umontpellier.fr (L.B.); jean.martinez@umontpellier.fr (J.M.); muriel.amblard@umontpellier.fr (M.A.)
2 ICGM, Univ Montpellier, CNRS, ENSCM, 34095 Montpellier, France
3 IRMB, Univ Montpellier, INSERM, CHU Montpellier, 34090 Montpellier, France; marie.maumus@inserm.fr
4 Bauerfeind France, IRMB, 34090 Montpellier, France
* Correspondence: daniele.noel@inserm.fr (D.N.); ahmad.mehdi@umontpellier.fr (A.M.); gilles.subra@umontpellier.fr (G.S.)
† These authors contributed equally to this work.

Abbreviations

ESI-MS, electrospray ionization mass spectrometry; HPLC, High Performance Liquid Chromatography; LC-MS, Tandem Liquid Chromatography/Mass Spectroscopy. Other abbreviations used were those recommended by the IUPAC-IUB Commission (Eur. J. Biochem. 1984, 138, 9-37).

Materials and method

All solvents and reagents were used as supplied. Anhydrous DMF was used and purchased from Acros. NaF was purchased from Acros. NMR solvents were obtained from Eurisotop. Isocyanatopropyl triethoxysilane (ICPTES) was obtained from TCI Europe. All amino acid derivatives and HATU (1-[Bis(dimethylamino)methylene]-1H-1,2,3-triazolo[4,5-b]pyridinium 3-oxid hexafluorophosphate were purchased from Iris Biotech GmbH (Marktredwitz, Germany). Amphispheres 40-RAM resin (Rink amide 40 (40% w/w PEG)) from Agilent Technologies (Santa-Clara, USA). Solvents were purchased from Carlo Erba reagents (Val de Reuil, France). Trifluoroacetic acid, 99%, was obtained from Acros Organics (New Jersey, USA). DPBS (DPBS - Dulbecco's Phosphate- Buffered Saline) buffer was purchased from Thermo Fischer (Waltham, USA). Other chemicals were obtained from Sigma- Aldrich (St. Louis, MO, USA). The polypropylene reaction vessels (syringes with frits) were purchased from Biotage (Uppsala, Sweden).

LC-MS analyses samples were prepared in an acetonitrile/water (50/50 v/v) mixture, containing 0.1% TFA. The LC-MS system consisted of a Waters Alliance 2695 HPLC, coupled to a Micromass (Manchester, UK) ZQ spectrometer (electrospray ionization mode, ESI+). Identification was carried out using a Merck Chromolith Speed rod C18, 25 × 4.6 mm reversed-phase column. A flow rate of 3 mL/min and a gradient of (0–100) % B over 2.5 min were used. Eluent A: water 0.1% HCO_2H; eluent B: acetonitrile 0.1% HCO_2H. Retention times (RT) are given in minutes. Positive ion electrospray mass spectra were acquired at a solvent flow rate of 200 mL/min. Nitrogen was used for both nebulizing and drying gas. The data were obtained in a scan mode ranging from 200 to 1600 m/z in 0.1 s intervals; 10 scans were summed up to get the final spectrum.

RP-preparative HPLC purification (for less than 2 g of crude product) was performed on a Gilson HPLC 2250, equipped with a C18 reversed-phase column. Standard conditions were eluent system A (water 0.1% TFA) and system B (acetonitrile 0.1% TFA). A flow rate of 50 mL/min and a gradient of (0-100) % B with adapted times were used, at a detection wavelength of 214 nm.

RP-preparative HPLC purifications (for more than 2 g of crude product) were performed on a NOVASEP system (NOVASEP LC 80.600.VE) equipped with a C18 reversed-phase Luna column (Phenomenex 10 μm, 250 × 50 mm) with a flow rate of 120 mL/min, by Luc Brunel (SynBio 3 platform).

Eluents were $H_2$0 1‰ TFA (A) and ACN 1‰ TFA (B). UV detection was adapted to the purified compound.

^{1}H NMR spectra were recorded on a Bruker Avance III HD 400 MHz spectrometer equipped with a BBFO probe and ^{29}Si and ^{13}C NMR spectra were recorded on a Bruker Advance III 500 MHz spectrometer equipped with a Helium BBO cryoprobe at the "Laboratoire de Mesures Physiques" of the University of Montpellier. ^{29}Si, ^{13}C and ^{1}H NMR spectra were recorded at 310 K in DMSO-d_6. Chemical shifts (δ) were reported in parts per million (ppm) using residual non-deuterated solvents as internal references (DMSO-d_6, δH = 2.50 ppm). Signals were indicated as s (singlet), d (doublet), t (triplet), q (quadruplet), dd (double doublet), m (multiplet). Coupling constants (J) were measured in Hertz.

Spectra were processed, visualized and analysed using Topspin 3.5 (Bruker Biospin).

Synthesis of hybrid collagen-like peptides (6M-2Si and 6M-1Si)

The FPPS methodology developed in our group (https://youtu.be/2aFQuPqsXCY) was applied to the SPPS synthesis of all peptides. All the stages of the FPPS were realized under vortex stirring at 450-650 rpm (depending of the volume). Synthesis were made with a Fmoc/tBu strategy. Stock solutions of all Fmoc-protected amino acids (Fmoc-Lys(Boc)-OH, Fmoc-Gly-OH, Fmoc-Hyp(tBu)-OH and Fmoc-Pro-OH) and HATU were prepared in DMF at 0.5 M.

Synthesis of Fmoc-Pro-Hyp(tBu)-Gly-OH

This synthesis was the only one that was not made on FPPS platform synthesis, but on a wheel for stirring because of the high volume of the reaction, on a 300 mL flash chromatography column used as a reactor to make the deprotection steps and a 1 L balloon to make the coupling steps. The synthesis was made on a 2.2-Cl-chlorotrityl resin (1.1 mmol/g, 20.0 g) in DMF. The first coupling reaction was performed using a Fmoc-Gly-OH (28.5 g, 4.5 eq) / DIEA (25.0 mL, 9 eq)/DMF (240 mL) mixture for 7 hours. The second coupling reaction was performed using a Fmoc-Hyp(tBu)-OH (24.0 g, 2.7 eq) / DIEA (20.6 mL, 5.4 eq) / HATU (21.7 g, 2.6 eq) /DMF (240.0 mL) mixture for 7 hours. The last coupling reaction was performed using a Fmoc-Pro-OH (31.2 g, 4.0 eq) / DIEA (30.9 mL, 8.0 eq) / HATU (32.40 g, 3.9 eq) /DMF (240 mL) mixture for 7 hours. The Fmoc removal steps were realized using a piperidine/DMF 20/80 v/v solution (200 mL) for 10 minutes and performed thrice. All washings were done with DMF twice, methanol once, DMF once, and DCM thrice (200 mL), after coupling steps and after deprotection steps. The peptide was cleaved from the resin with the mixture DCM/HFIP (80/20 v/v/v) to keep the tBu protection (2x1h30), evaporated under reduced pressure, recovered by precipitation in diethyl ether, then taken up in ACN/H_2O 50/50 v/v mixture and freeze-dried. The crude peptide was solubilized in H_2O/ACN 98/2 v/v mixture with 1‰ TFA and purified by RP-preparative HPLC. The purification was performed on the NOVASEP system at 235 nm. Eluents were H_2O 1‰ TFA (A) and ACN 1‰ TFA (B). The purification gradient started at 0% of B and increased from 0 to 50% of B in 10 min and then from 50 to 90% in 15 min. Collected fractions were concentrated and freeze-dried to yield the pure tripeptide with ≈ 17 g (Figure S1).

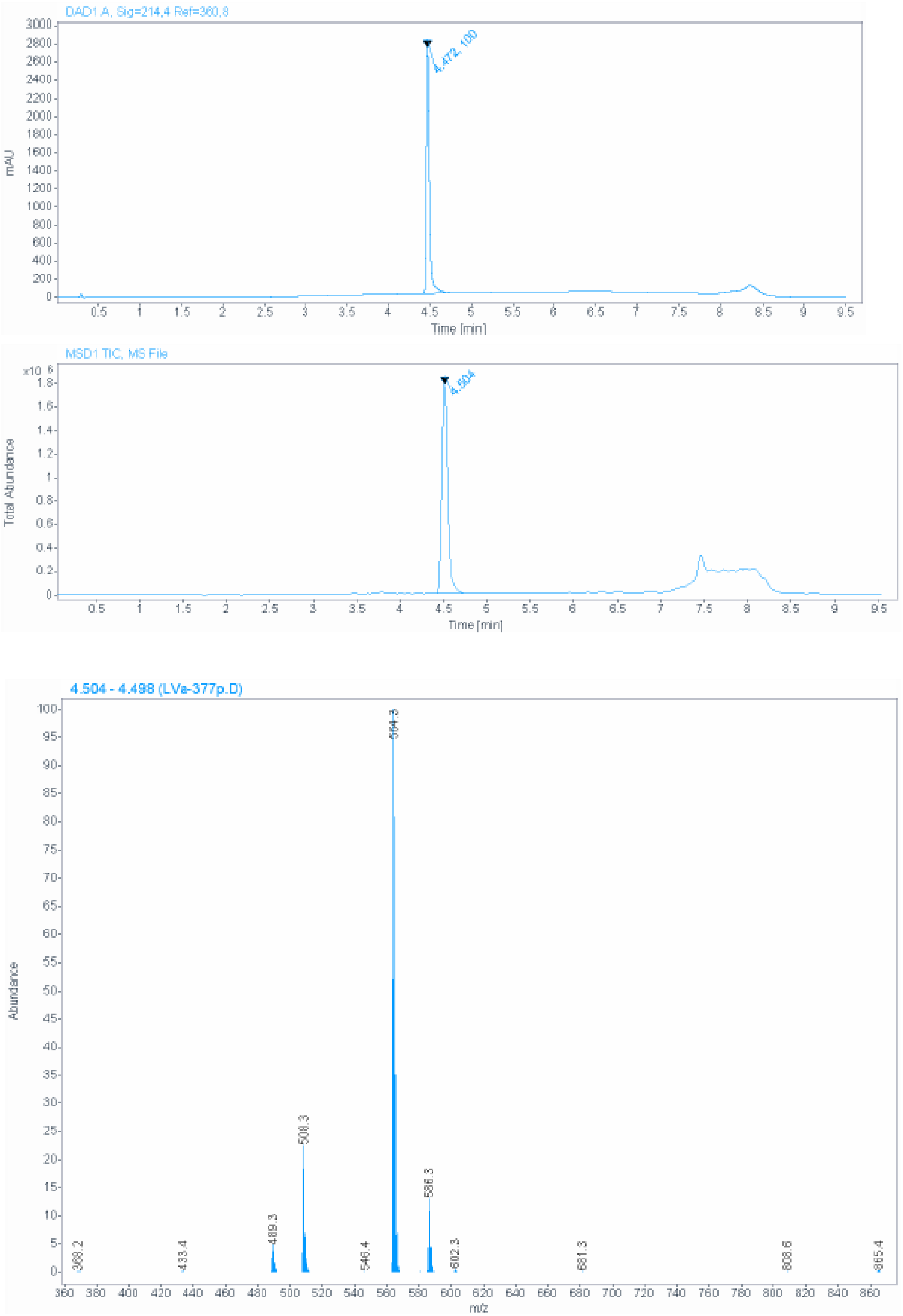

Figure S1. LC-MS spectra of Fmoc-Pro-Hyp(tBu)-Gly-OH. LC-MS (ESI+): tR = 4.504 min, m/z 564 $[M+H]^+$; m/z 586 $[M+Na]^+$.

Synthesis of Ac-Lys-(Pro-Hyp-Gly)$_6$-Lys-NH$_2$, 2 TFA (6M2K)

Synthesis was made by FPPS using the AmphiSpheres 40 RAM (0.15 mmol/g, 3.870 g) resin in DMF. The first coupling reaction was performed using a Fmoc-Lys(Boc)-OH (1.370 g, 5.0 eq) / DIEA (1.019 mL, 10.0 eq)) / HATU (1.100 g, 5.0 eq) / DMF (5.0 mL) mixture overnight. The second coupling reaction was performed using a Fmoc-Pro-Hyp(tBu)-Gly-OH (0.616 g, 1.9 eq) / DIEA (0.406 mL, 4.0 eq)) / HATU (0.414 g, 1.9 eq) / DMF (5.0 mL) mixture for 6 hours. The third coupling reaction was performed using a Fmoc-Pro-Hyp(tBu)-Gly-OH (0.567 g, 1.75 eq) / DIEA (0.374 mL, 3.7 eq)) / HATU (0.381 g, 1.75 eq) / DMF (5.0 mL) mixture overnight. The fourth coupling reaction was performed using a Fmoc-Pro-Hyp(tBu)-Gly-OH (0.486 g, 1.5 eq) / DIEA (0.321 mL, 3 .0 eq)) / HATU (0.327 g, 1.5 eq) / DMF (5.0 mL) mixture for 6 hours. The fifth coupling reaction was the same than the fourth but performed overnight. The sixth coupling reaction was the same than the fourth. The seventh reaction was the same than the fifth. The last coupling reaction was performed using a Fmoc-Lys(Boc)-OH (1.370 g, 5.0 eq) / DIEA (0.509 mL, 10.0 eq)) / HATU (1.100 g, 5.0 eq) / DMF (5.0 mL) mixture for 20 minutes twice with a washing step with DMF in between. The Fmoc removal steps were realized using a piperidine/DMF 20/80 v/v solution (20 mL) for 10 minutes and performed thrice. All washings were done with DMF thrice, methanol once, DMF once, and DCM twice (20 mL), after coupling steps and after deprotection steps. N-Terminal acetylation was performed with 20 % Ac_2O in DMF and DIEA (1 %) for 15 minutes twice. The peptide was cleaved from the resin with pure TFA (2x1h30), concentrated under reduced pressure, recovered by precipitation in diethyl ether, then taken up in ACN/H_2O 50/50 v/v mixture and freeze-dried.

The crude peptide was solubilized in H_2O/ACN 98/2 v/v mixture with 1‰ TFA and purified by RP-preparative HPLC. The purification was performed on a NOVASEP system at 225 nm. Eluents were H_2O 1‰ TFA (A) and ACN 1‰ TFA (B). The purification gradient started with 5 minutes at 0 % of B and then increased from 0 to 20 % of B in 25 min. Collected fractions were concentrated and freeze-dried to yield 600 mg of the pure **6M2K** peptide as a TFA salt (white powder) with 48 % yield (Figure S2).

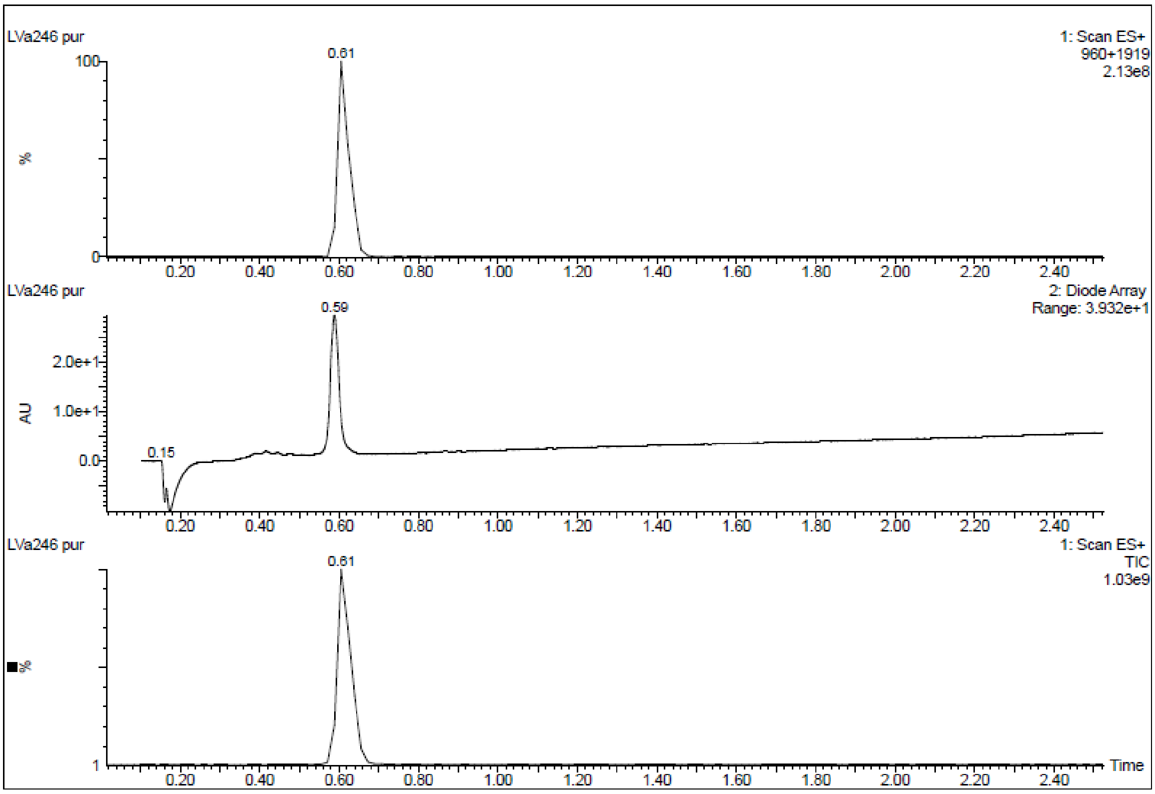

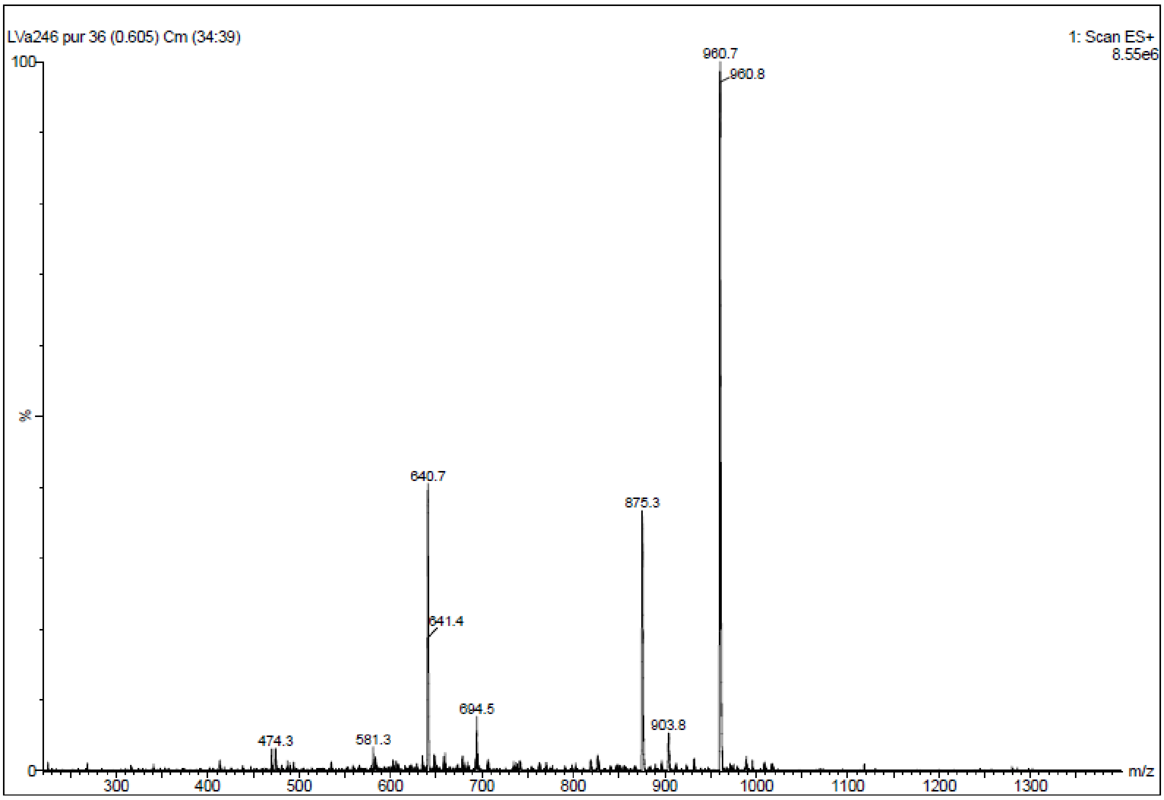

Figure S2. LC-MS spectra of **6M2K**. LC-MS (ESI+): tR = 0.59 min, m/z 960.7 $[M+2H]^{2+}$; m/z 640.7 $[M+3H]^{3+.}$

Silylation of Ac-Lys-(Pro-Hyp-Gly)$_6$-Lys-NH$_2$ (6M-2Si)

6M2K (1.000 g, 0.466 mmol) was solubilized in anhydrous DMF (7.0 mL). DIEA (364 µL, 2.07 mmol, 4.4 eq) and ICPTES (459 µL, 1.76 mmol, 3.8 eq) were added under stirring and inert atmosphere (argon). The solution was stirred at RT for 1h30. The product was precipitated by addition of diethyl ether and the obtained white solid was washed 6 times with diethyl ether and vacuum-dried. 1.12 g of **6M-2Si** was quantitatively obtained (Figure S3).

^{1}H NMR spectrum shows silylation with triethoxysilyl moieties signals (Figure S4) but also other spectra (Figure S5 and Figure S6).

This silylated peptide could be kept under inert atmosphere in freezer for maximum 4 days until condensation occurs.

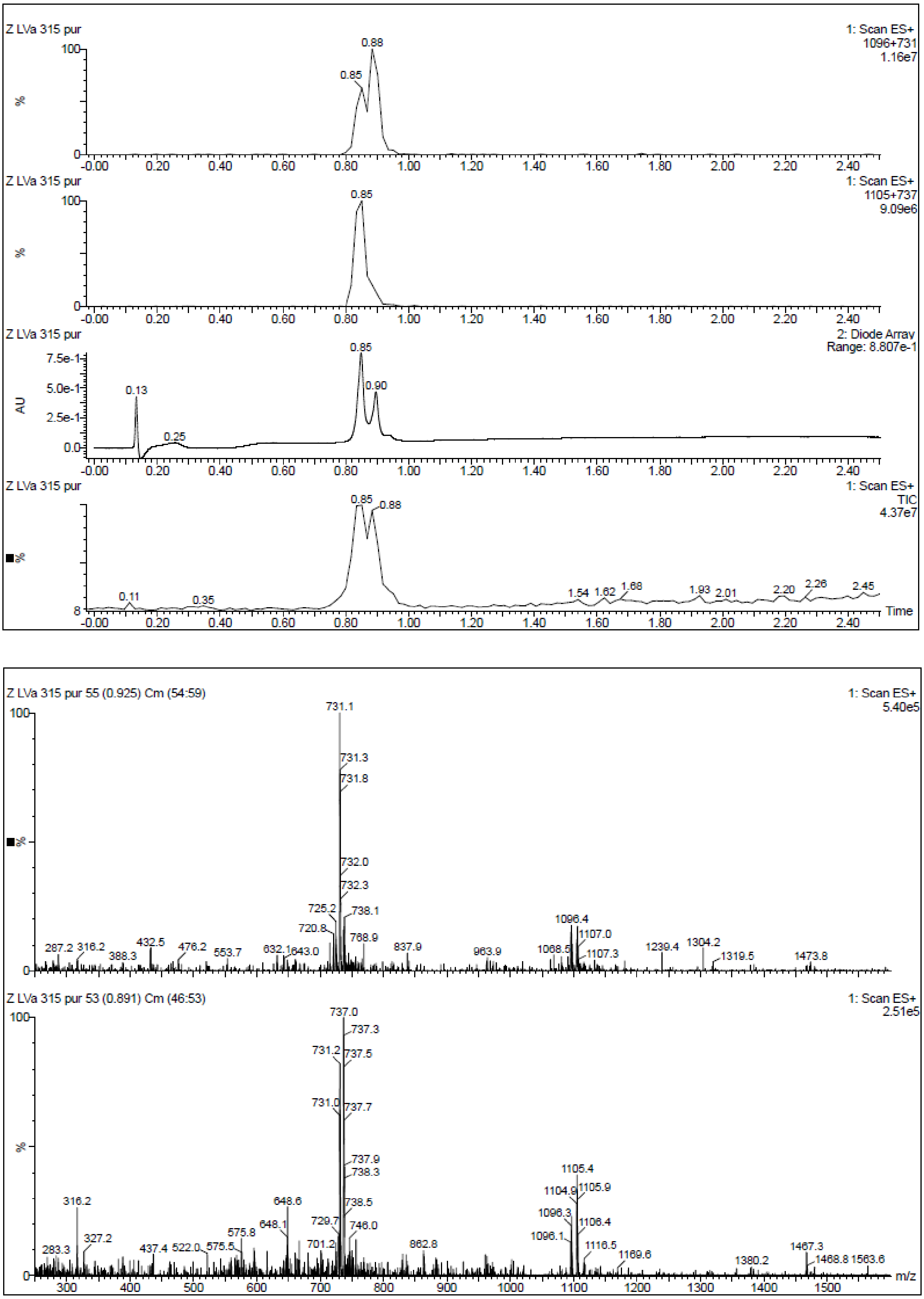

Figure S3. LC-MS spectra of **6M-2Si**. LC-MS (ESI+): The peptide is always analysed hydrolyzed due to acidic water solvent. Loss of water molecule is due to condensation of the peptide. tR = 0.85-0.90 min, m/z 1105.4 [M hydrolyzed – 2 H_2O + 2H]$^{2+}$; m/z 1096.4 [M hydrolyzed – 3 H_2O + 2H]$^{2+}$; m/z 737.0 [M hydrolyzed – 2 H_2O + 3H]$^{3+}$; m/z 731.0 [M hydrolyzed – 3 H_2O + 3H]$^{3+}$.

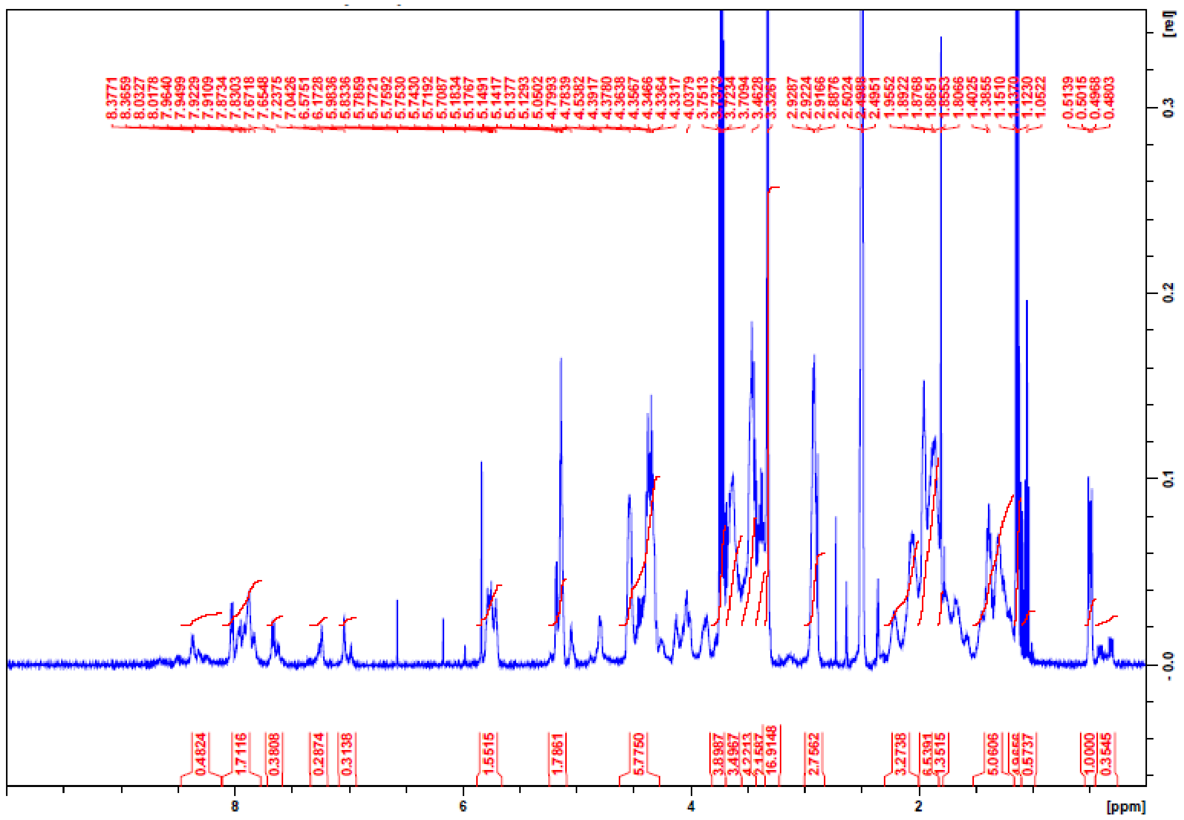

Figure S4. ^{1}H NMR spectrum of **6M-2Si** in DMSO-d_6 (400 MHz). ^{1}H NMR (400 MHz, DMSO-d_6) δ 3.73 (q) CH_2 of triethoxysilyl, 1.14 (t) CH_3 of triethoxysilyl, 1.07 (t) CH_3 of EtOH from triethoxysilyl hydrolysis, 0.50 (t) CH_2 in α position of triethoxysilyl.

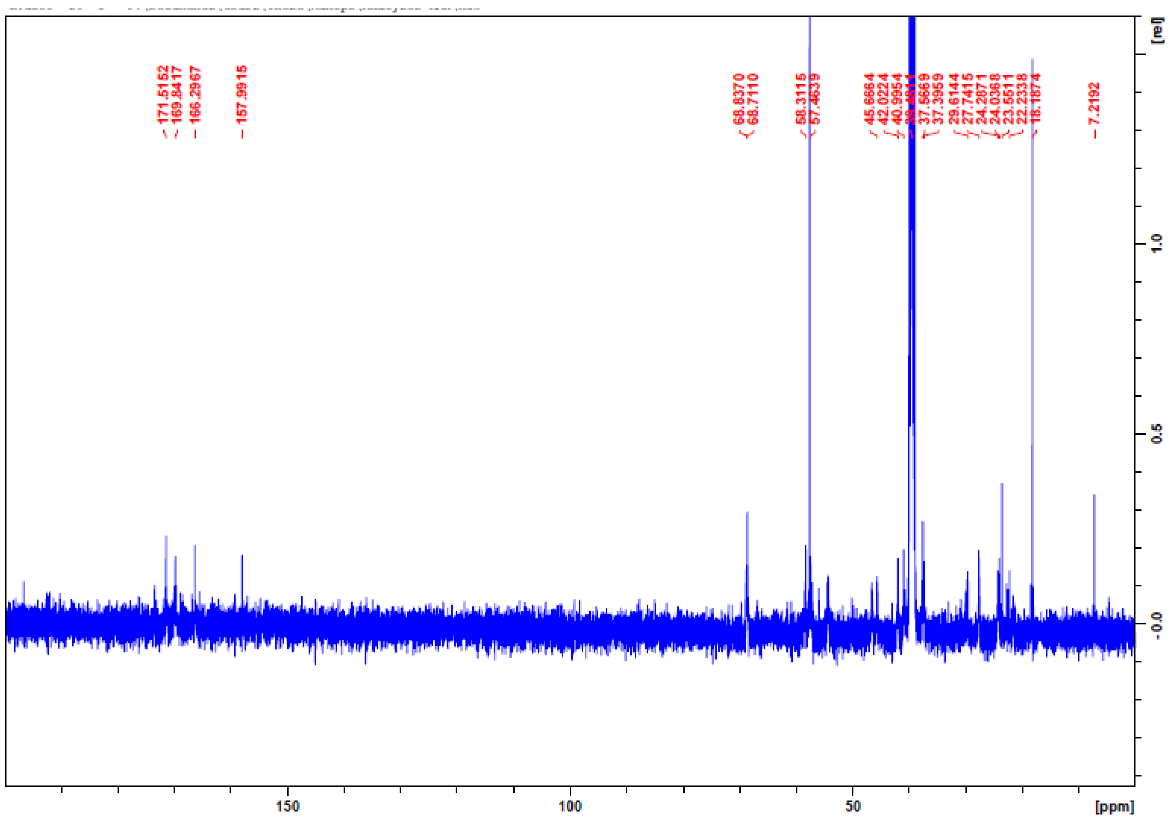

Figure S5. ^{13}C NMR spectrum of **6M-2Si** in DMSO-d_6 (100.6 MHz).

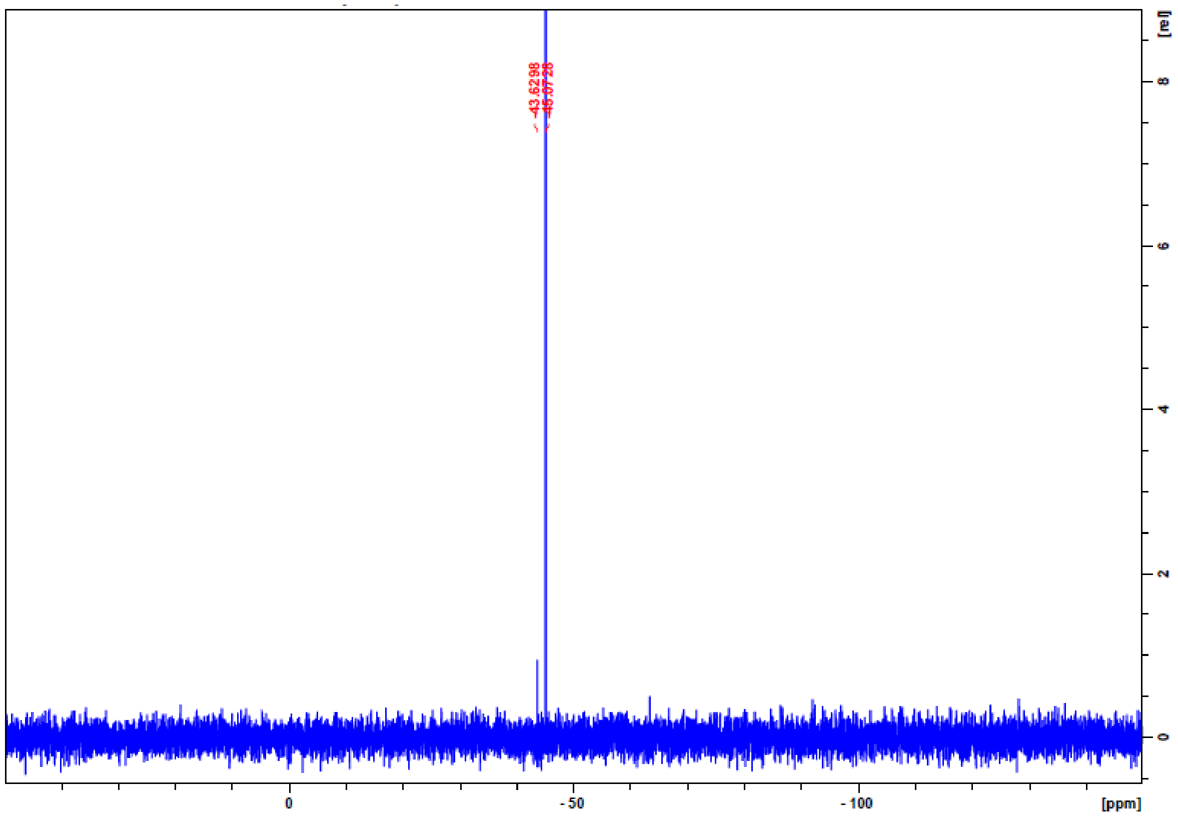

Figure S6. ^{29}Si NMR spectrum of **6M-2Si** in DMSO-d_6 (99 MHz). ^{29}Si NMR (99 MHz, DMSO-d_6) δ -45.1 for -Si(OEt)$_3$ moities.

Synthesis of Ac-(Pro-Hyp-Gly)$_6$-Lys-NH$_2$, 1 TFA (6M1K)

Synthesis by FPPS was made using the AmphiSpheres 40 RAM (0.27 mmol/g, 1.200 g) resin in DMF. The first coupling reaction was performed using a Fmoc-Lys(Boc)-OH (0.759 g, 5.0 eq) / DIEA (0.569 mL, 10.0 eq)) / HATU (0.616 g, 5.0 eq) / DMF (5.0 mL) mixture overnight. The second coupling reaction was performed using a Fmoc-Pro-Hyp(tBu)-Gly-OH (0.344 g, 1.9 eq) / DIEA (0.213 mL, 4.0 eq)) / HATU (0.226 g, 1.9 eq) / DMF (4.0 mL) mixture for 4 hours. The third coupling reaction was performed using a Fmoc-Pro-Hyp(tBu)-Gly-OH (0.317 g, 1.75 eq) / DIEA (0.196 mL, 3.7 eq)) / HATU (0.208 g, 1.75 eq) / DMF (4 mL) mixture for 4 hours. The fourth coupling reaction was performed using a Fmoc-Pro-Hyp(tBu)-Gly-OH (0.272 g, 1.5 eq) / DIEA (0.168 mL, 3.0 eq)) / HATU (0.178 g, 1.5 eq) / DMF (4 mL) mixture overnight. The fifth and sixth coupling reactions were the same than the fourth but performed in 4 hours. The seventh coupling reaction was the same than the fourth. The last coupling reaction was performed using a Fmoc-Lys(Boc)-OH (0.759 g, 5.0 eq) / DIEA (0.569 mL, 10.0 eq)) / HATU (0.616 g, 5.0 eq) / DMF (4.0 mL) mixture for 20 minutes twice with a washing step with DMF in between. The Fmoc removal steps were realized using a piperidine/DMF 20/80 v/v solution (20 mL) for 10 minutes and performed thrice. All washings were done with DMF thrice, methanol once, DMF once, and DCM twice (20 mL), after coupling steps and after deprotection steps. N-Terminal acetylation was performed with 20 % Ac_2O in DMF and DIEA (1 %) for 15 minutes twice. The peptide was cleaved from the resin with pure TFA (2x1h30), concentrated under reduced pressure, recovered by precipitation in diethyl ether, then taken up in ACN/H_2O 50/50 v/v mixture and freeze-dried.

The crude peptide was solubilized in H_2O with 1‰ TFA and purified in three injections by RP-preparative HPLC. The purification was performed on the Gilson system. Eluents were H_2O 1‰ TFA (A) and ACN 1‰ TFA (B). The purification gradient started with 5 minutes at 0% of B, then increased from 0 to 10% of B in 5 min, and then from 10 to 15 % of B in 15 min. Collected fractions were concentrated and freeze-dried to yield 322 mg of the pure **6M1K** peptide as a TFA salt (white powder) with 49 % yield (Figure S7).

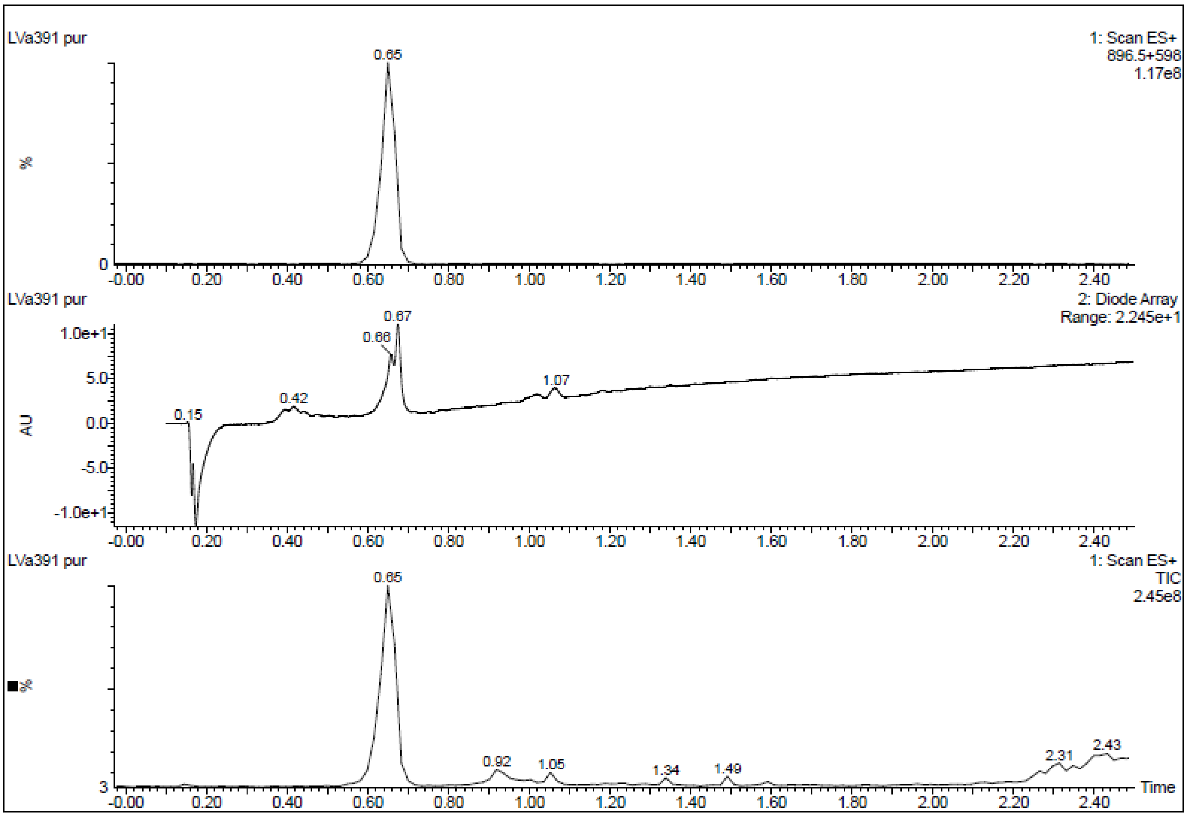

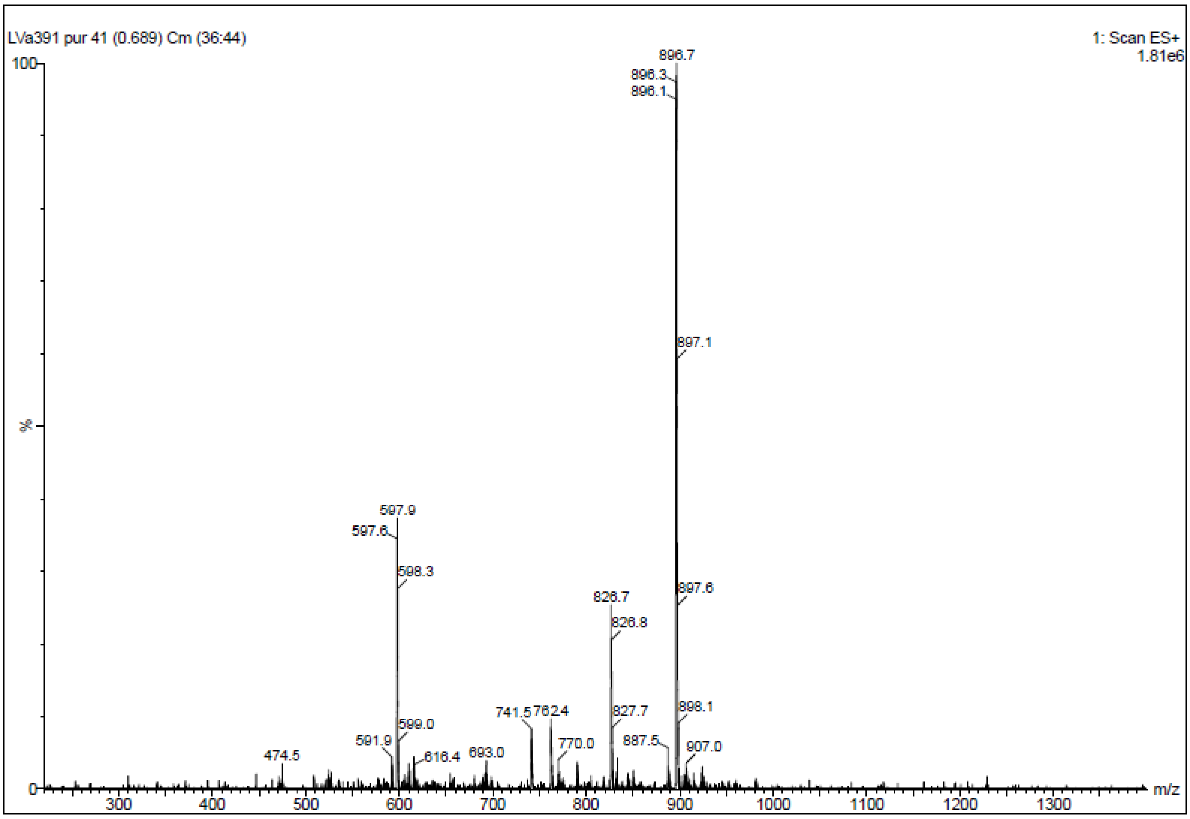

Figure S7. LC-MS spectrum of **6M1K**. LC-MS (ESI+): tR = 0.65 min, m/z 896.7 $[M+2H]^{2+}$; m/z 597.9 $[M+3H]^{3+}$.

Silylation of Ac-(Pro-Hyp-Gly)$_6$-Lys-NH$_2$ (6M-1Si)

6M1K (0.040 g, 0.021 mmol) was solubilized in anhydrous DMF (0.225 mL). DIEA (10 µL, 0.058 mmol, 2.8 eq) and ICPTES (10 µL, 0.040 mmol, 1.9 eq) were added under stirring and argon atmosphere. The solution was stirred at RT for 1h30. The product was precipitated by addition of diethyl ether and the obtained white solid was washed 3 times with diethyl ether and vacuum-dried. 40 mg of **6M-1Si** was quantitatively obtained (Figure S8).

^{1}H NMR spectrum shows silylation with triethoxysilyl moieties signals (Figure S9) but also other spectra (Figure S10 and Figure S11).

This silylated peptide could be kept under inert atmosphere in freezer for maximum 4 days until condensation occurs.

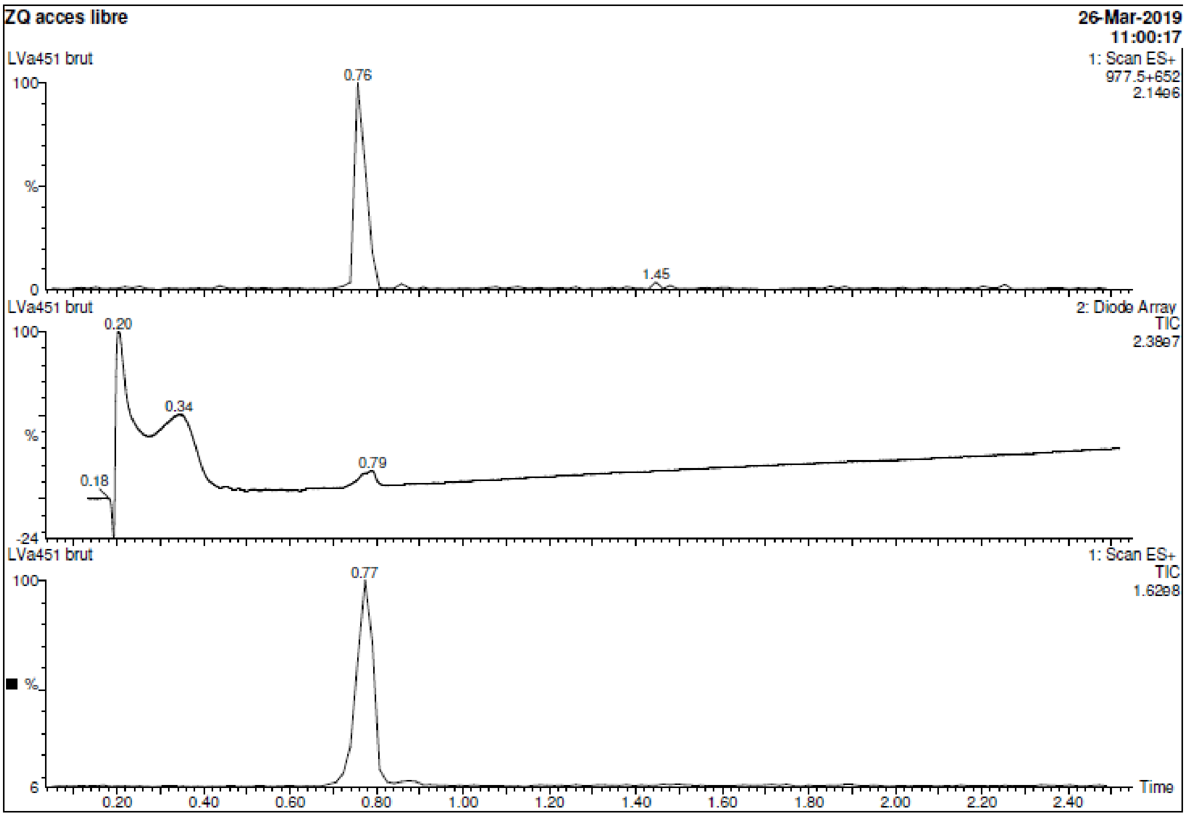

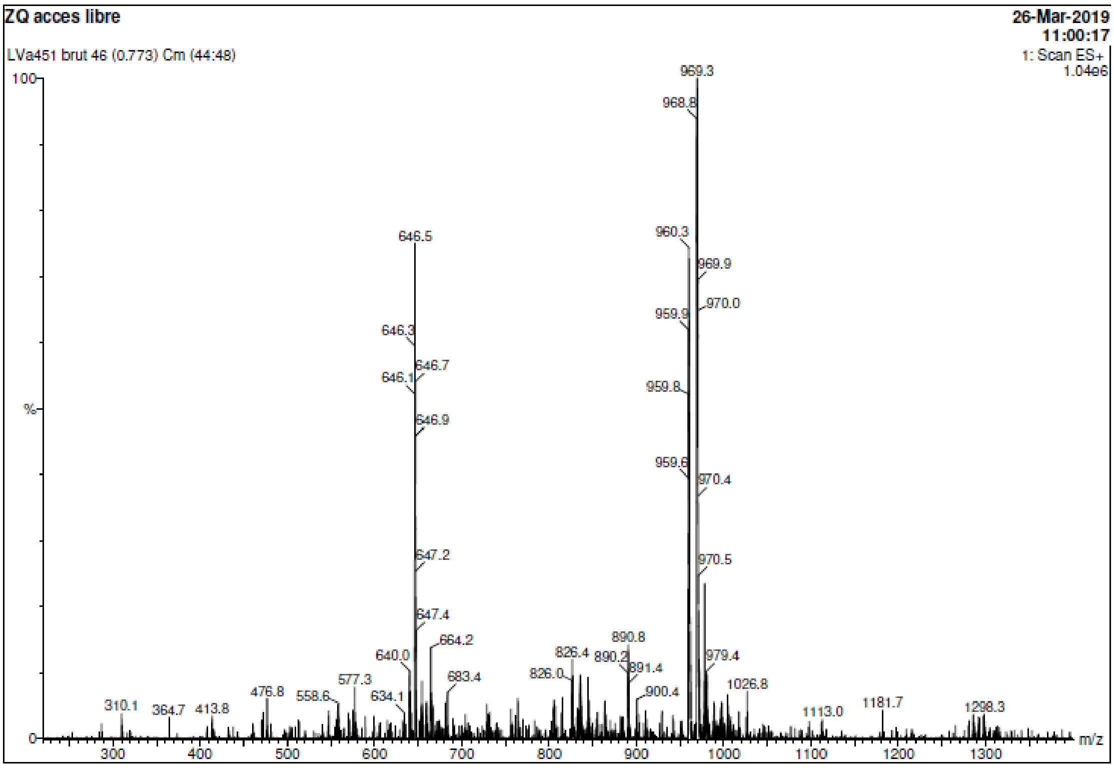

Figure S8. LC-MS spectra of **6M-1Si** (crude product). LC-MS (ESI+): The peptide is always analysed hydrolyzed due to acidic water solvent. Loss of water molecule is due to condensation of the peptide. tR=0.20 and 0.34 min DMF and DIEA of the crude product. tR = 0.68 min, m/z 969.3 [M hydrolyzed – 2 H_2O + 2H]$^{2+}$; m/z 960.3 [M hydrolyzed – 3 H_2O + 2H]$^{2+}$; m/z 646.5 [M hydrolyzed – 2 H_2O + 3H]$^{3+}$.

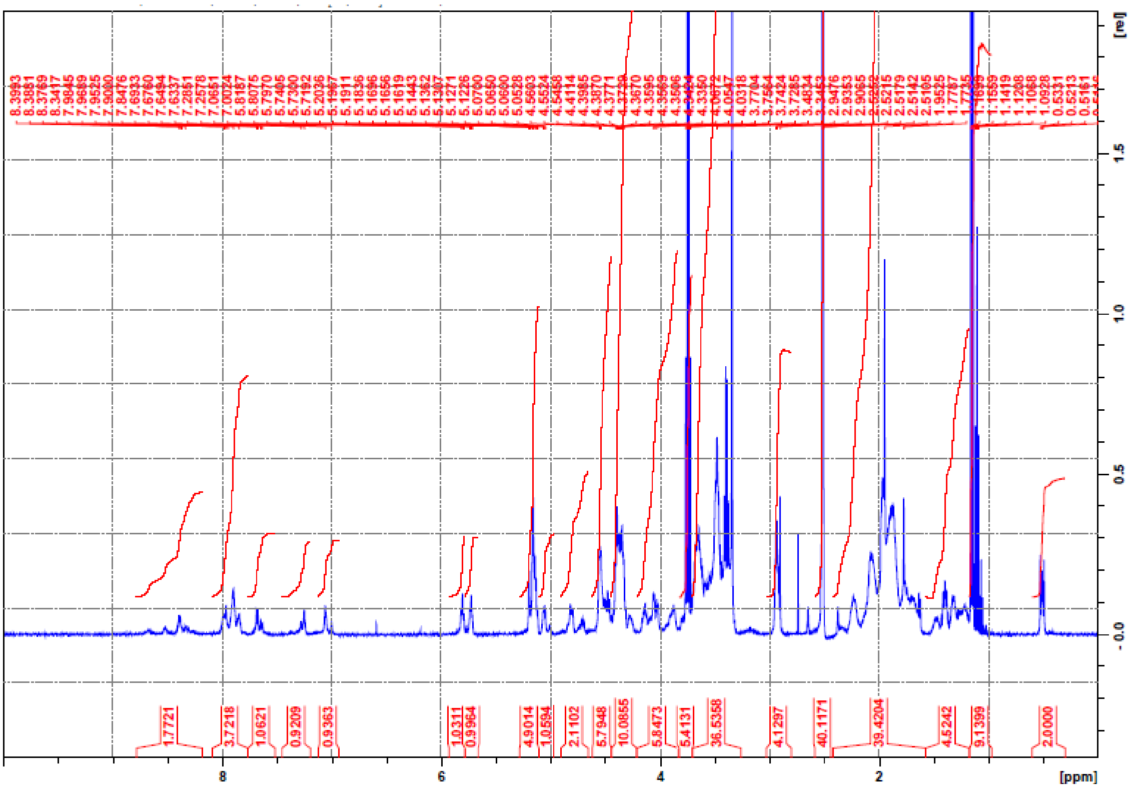

Figure S9. ^{1}H NMR spectrum of **6M-1Si** in DMSO-d$_6$ (400 MHz). ^{1}H NMR (400 MHz, DMSO-d$_6$) δ 3.73 (q) CH_2 of triethoxysilyl, 1.14 (t) CH_3 of triethoxysilyl, 1.07 (t) CH_3 of EtOH from triethoxysilyl hydrolysis, 0.50 (t) CH_2 in α position of triethoxysilyl.

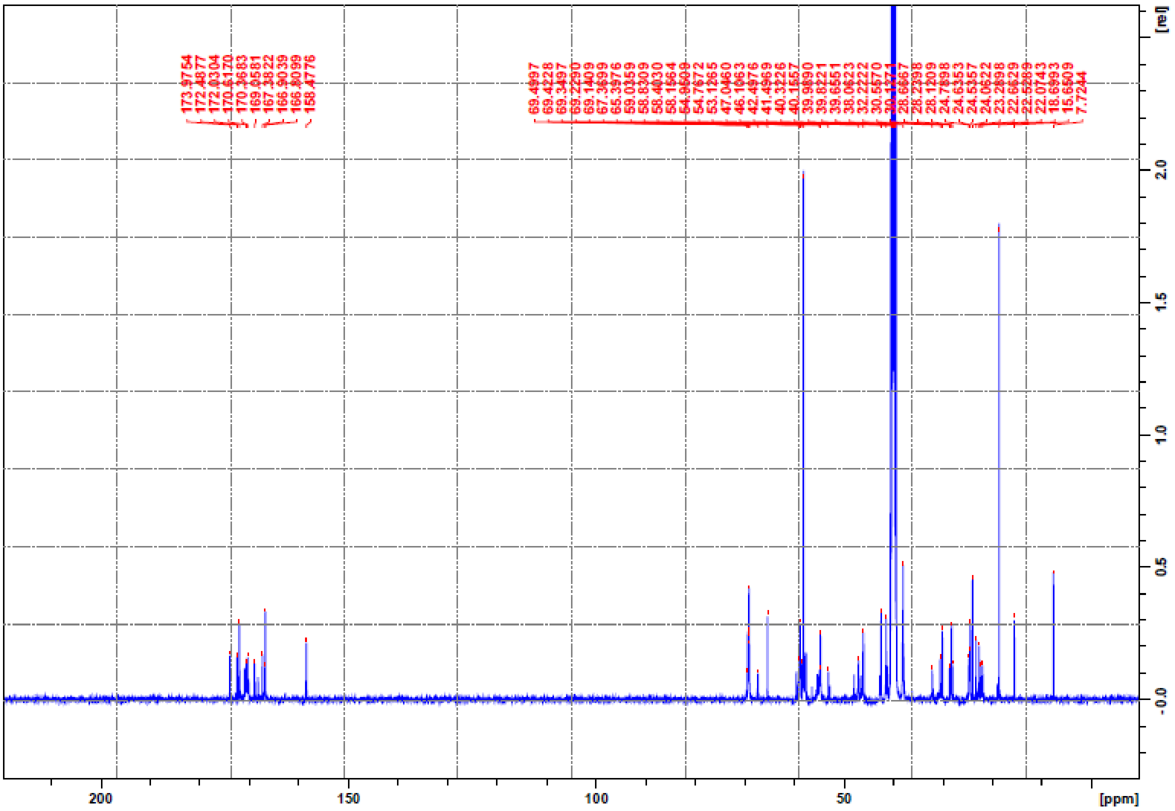

Figure S10. ^{13}C NMR spectrum of **6M-1Si** in DMSO-d_6 (100.6 MHz).

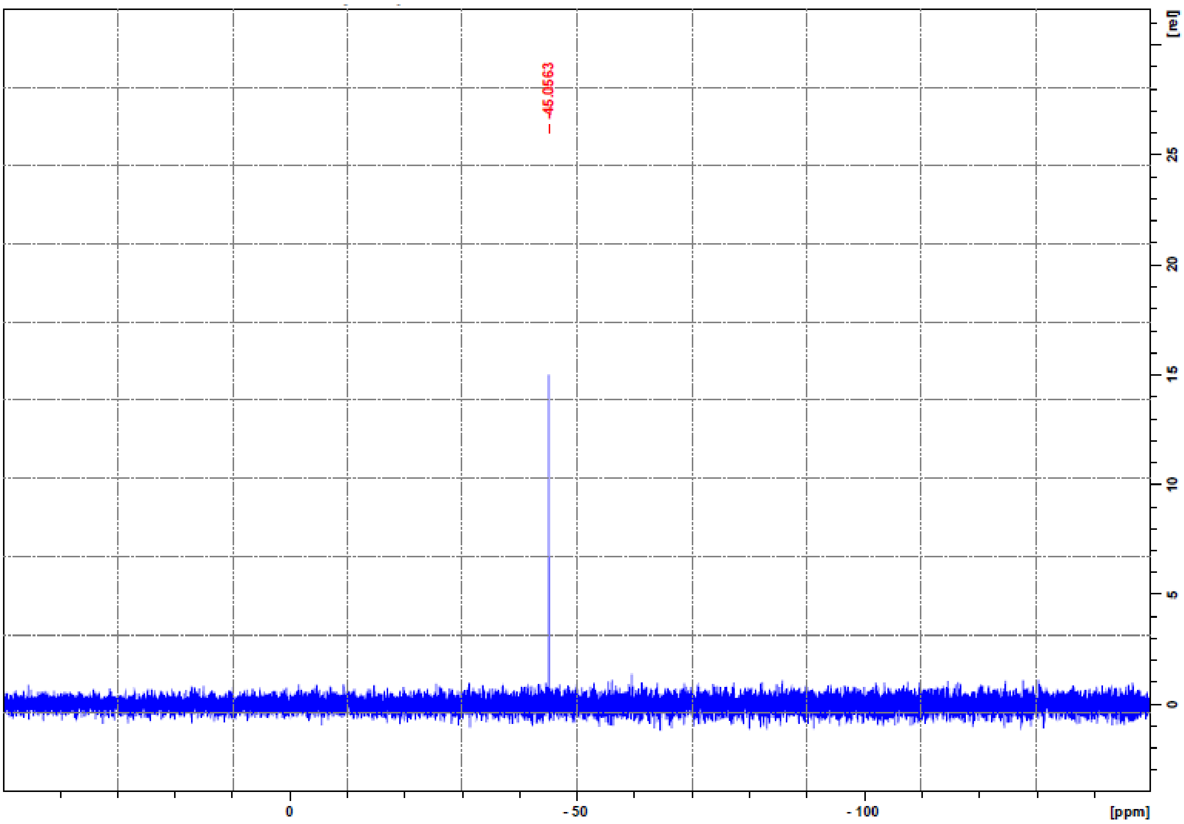

Figure S11. ^{29}Si NMR spectrum of **6M-1Si** in DMSO-d_6 (99 MHz). ^{29}Si NMR (99 MHz, DMSO-d_6) δ -45.1 for -Si(OEt)$_3$ moities.

Physico-chemical characterisation of peptide-based hydrogels

Rheological studies

All measurement were performed on a HR-2 rheometer from TA Instrument.

The macro-indentation experiments were performed on a formed hydrogel (1.20 mL) poured in a 12 wells plate after 6 days of aging at 37 °C in humid atmosphere. The indentation test was performed with a duralumin cylinder connected at one end to the stress-controlled rheometer, and presenting a circular flat surface on the other end (diameter 10 mm). The speed of descent was set to 0.1 mm/s until to reach a 200 µm gap (≈92 % of strain). The normal force sensor of the rheometer was used as a force gauge to determine the stress-strain relation during the sample indentation. The maximum normal stress (σ_{max}) and compressive strain (ε_{max}) could be read when the hydrogel cracked under compression, hydrogel when the curve σ =f(ε) fall due to the rupture (**Figure S12**). In the first moments of the deformation (under 5 % of deformation), the linear low-strain regime provides a way to define an apparent elastic modulus ($E^* = \delta\sigma/\delta\varepsilon$), and after calculation, the Young's modulus E and then the storage modulus by calculation from it. At last, the mesh size can be calculated (Figure S12, equations 1-3). This value gives information about the rigidity of the hydrogel. The experiment was made in duplicate. A second pic of deformation could be attributed to the detachment of the gel from the well.

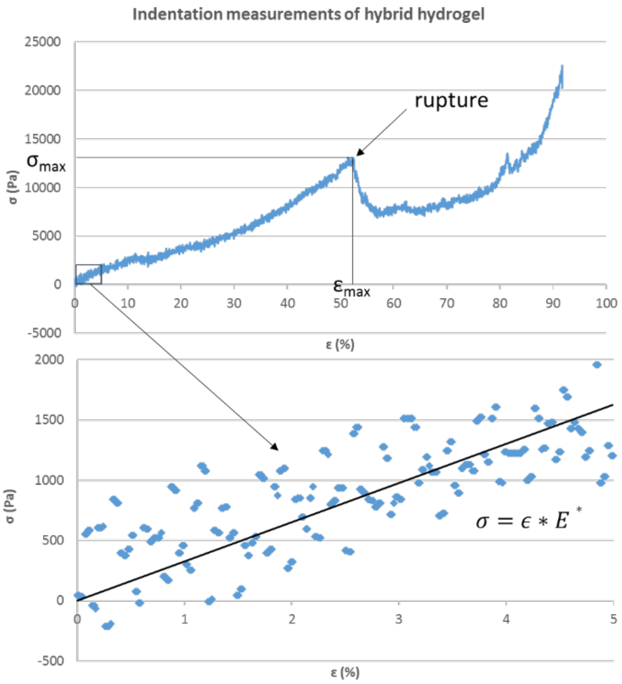

Equation 1: $E = (1-\nu^2)\frac{r}{e}\left(\frac{\pi}{2\kappa}\right)E^*$ Young's modulus

Equation 2: $G' = \frac{E}{2(1+\nu)}$ storage modulus

Equation 3: $\xi = \left(\frac{KT}{G'}\right)^{\frac{1}{3}}$ mesh size

Symbol	Name	Value
ν	Poisson coefficient	0.5
r	Radius of the indenter	5 mm
e	Thickness of the gel disk	≈ 2.4 mm
κ	Numerical correction related to geometrical effects in relation with the variables, e and ν	2
K	Boltzmann's constant	1,38 x 10^{-23} J K^{-1}
T	Temperature	295 K

Figure S12. Example of indentation measurements on a hybrid hydrogel and calculations of Young's modulus, storage modulus and mesh size.

Swelling experiments

Hydrogels were weighted and freeze-dried. The obtained foams were weighted and swollen into water (pH 7.4) for 8 or 24 hours before weighted wet (a blue unwoven paper was used to absorb the surfacing water). All measurements were made in three samples for triplicates. Water uptake and mesh size were calculated from weights obtained.

For each calculation of mesh size, some approximations had to be made. The dry polymer density, the solvent-polymer interaction parameter (Flory-Huggins) and the characteristic ratio of the polymer were taken from literature for collagen.[1,2] For hybrid collagen-like peptide we made the approximation that these values were the same than the one of gelatine we can found in literature.[3]

Table S1. Symbols names and values used for mesh size calculation. The first value is the one used for **6M-2Si** and the second for collagen.

Symbol	Name	Value
ϱ_p	Polymer volumetric mass	1.35 and 1.41 g/cm^3
ϱ_s	Solvent volumetric mass	1 g/cm^3
M_n	Number average molecular weight of the polymer before cross-linking	2412.3 and 300000 g/mol
$\tilde{v}$	Specific volume of the polymer (=1/$\varrho_{polymer}$)	Calculated from ϱ_p
V_1	Molar volume of the swelling agent	18 cm^3/ mol
χ_1	Flory polymer-water interaction parameter	0.8 and 0.57
l	Average bond length	0.143 nm
C_n	Polymer characteristic ratio	8.3 and 9.0
M_{UR}	Molecular weight of the repeating unit	267 g/mol

The following equation are adapted from previous published paper.[4] First, mass (Equation 1) and volume swelling degree (Equation 4) (respectively Qm and Qv) and volume fraction at equilibrium of swelling (ν_2) (Equation 5) were determined with values obtained in swelling studies (see following section for description of experiments). Note that swelling rate is different from swelling percentage.

Equation 4: $Q_m = \frac{m(wet)}{m(dried)}$

Equation 5: $Q_v = 1 + \frac{\rho_p}{\rho_s}(Q_m - 1)$

Equation 6: $\nu_2 = \frac{1}{Q_v}$

Then, using equation 7, the number average molecular weight between cross-links (M_c), was calculated from the equilibrium swelling data.

Equation 7: $\frac{1}{M_c} = \frac{2}{M_n} - \frac{(\frac{\tilde{v}}{V_1})(\ln(1-\nu_2) + \nu_2 + \chi_1 \nu_2^2)}{\nu_2{}^{1/3} - (\frac{\nu_2}{2})}$

The M_c value is the starting point for the estimation of the end-to-end distance of the unperturbed (solvent-free) state ($(\overline{r_0{}^2})^{1/2}$) (Equation 8)

Equation 8: $(\overline{r_0{}^2})^{1/2} = l C_n{}^{1/2} n^{1/2}$

With n: number of bonds in the cross-link (=2M_c/M_{UR})

Finally, the mesh size (ξ) was determined from Equation 9.

Equation 9: $\xi = \nu_2{}^{-1/3} \times (\overline{r_0{}^2})^{1/2}$

Biological evaluation of peptide-based hydrogels

Human mesenchymal stromal cells

Human mesenchymal stromal cells (hMSCs) were isolated from patients undergoing a total knee arthroplasty as previously described (Ruiz *et al.*, *Osteoarthritis cartilage*, 2019). Briefly, hMSCs were cultured in proliferative medium (αMEM (Lonza) supplemented with 10% foetal calf serum, 1% penicillin-streptomycin, 1% glutamine and 1ng/mL of basic Fibroblast Growth Factor (Cellgenix) at 37 °C in a humidified 5 % CO_2 atmosphere. hMSCs were characterized by cell surface markers ($CD90^+$/$CD73^+$/$CD105^+$/$CD45^-$) and by their tripotential differentiation abilities as previously described (Maumus et al, Stem Cell Research 2013). They were used at passage 2 or 3 for cell viability and chondrogenesis assessment.

Before each hydrogel preparation for biological evaluation, ^{1}H NMR analysis in DMSO d_6 was performed on each hybrid peptide to prevent that no residual solvent remained (DMF at 7.95, 2.89 and 2.73 ppm, and diethyl ether at 1.09 and 3.38 ppm).

Cell viability and chondrogenesis evaluation of cells embedded within hybrid hydrogels

Hybrid hydrogels with desired compositions were prepared in DPBS with 0.1 mg/mL NaF and 10 mg/mL glycine. The hMSCs were added to the hybrid gel solution at 10^6 cells/mL. These solutions were maintained under gentle agitation for few hours until a slight increase of the viscosity was reached visually. For cell viability and chondrogenesis experiments, 50 or 400 µL of hybrid hydrogels were distributed, respectively, in 96 or 24 wells of Ultra Low Adhesion (ULA) plates (Corning). After 24h

gelation at 37 °C, proliferative or chondrogenic medium (DMEM high glucose, 1 % penicillin-streptomycin, 1 mM sodium pyruvate, 170 µM 2-phosphate ascorbic acid, 350 µM proline, 100 nM dexamethasone, Insulin-Transferrin-Selenium and TGF-β3 (10 ng/mL, R&D Systems)) was added on top of hydrogels and culture plates were incubated in humidified chamber at 37 °C. The medium was changed every three days.

Cell viability was evaluated after 1, 7 or 21 days, with the LIVE/DEAD cell viability assay (Invitrogen, ref R37601) following manufacturer's instructions, and cells were imaged by confocal microscopy (Leica SP8 microscope). Cell number contained in hydrogels was evaluated by DNA quantification using the Cyquant cell proliferation assay (Thermofisher) following manufacturer's instructions. Cell number was normalized to 100% at day 0.

Chondrogenic differentiation was evaluated after 21 days of culture, by quantification of chondrogenic marker expression (*SOX9*, *COL2A1*, *ACAN*, *COL10*) by RT-qPCR. Positive control was hMSCs differentiated in micropellet as described (Dusfour, 2020, 110808) (data not shown), while negative controls were non-differentiated hMSCs at day 0 or hMSCs in hydrogels and cultured in incomplete chondrogenic medium (without TGF-β3).

References

1. P. Jean, F. M. Oth, *Colloid Polym. Sci.* **1960**, *168*, 49–56.
2. J. D. Bronzino, *The Biomedical Engineering Handbook 1*, Springer Science & Business Media, **2000**.
3. G. Wypych, in *Handb. Polym.*, Elsevier, **2016**, pp. 149–150.
4. T. Canal, N. A. Peppas, *J. Biomed. Mater. Res. A* **1989**, *23*, 1183–1193.
